# Smyd3, a histone methyltransferase, modulates the growth and differentiation of human cardiac stem cells

**DOI:** 10.1186/1471-2164-15-S2-P69

**Published:** 2014-04-02

**Authors:** Narasimman Gurusamy, Junghyun Kim, Toru Hosoda, Gauthaman Karunakaran, Mubarak Al Gahtany, Annarosa Leri

**Affiliations:** 1 Department of Pharmacology, College of Pharmacy, King Khalid University, Abha, Kingdom of Saudi Arabia; 2 Department of Anesthesia and Medicine, Brigham and Women's Hospital, Harvard Medical School, Boston, MA, USA

## Background

The phenotypic properties of embryonic and adult stem cells are largely mediated by epigenetic modifications which control stemness, multipotentiality and fate specification. In this study, our objective was to identify the epigenetic mechanism regulating the growth and differentiation of human cardiac stem cells (hCSCs).

## Materials and methods

Discarded surgical specimens were enzymatically digested and c-kit-positive hCSCs were sorted by FACS.

## Results

Genome-wide analysis of histone modifications documented that adult hCSCs are characterized by a bivalent chromatin configuration similar to that of embryonic stem cells with repressive and activating marks at lysine residues of histones H3 and H4. The high levels of di- (H3K4me2) and tri-methylation (H3K4me3) at lysine 4 of histone H3 prompted us to test the function of histone methyltransferase Smyd3, which is downregulated by the non-functional mutation in the c-kit receptor, in hCSCs. Inhibition of Smyd3 by siRNA strategy decreased the fraction (60%) of cycling hCSCs and increased apoptosis (2.5-fold). Attenuation of hCSC growth was coupled with a decline in the expression of telomerase, hTERT, and the myocyte specific transcription factors hNkx2.5 and hGata6. By immunolabeling, Smyd3 co-localized with hTERT, hNkx2.5 and hGATA6 in hCSC nuclei. Conversely, transfection of hCSCs with a plasmid carrying Smyd3 was coupled with upregulation of hTERT and hNkx2.5. Chromatin immunoprecipitation assay show that Smyd3 binds to hTERT, hGata6 and hNkx2.5 promoter regions that contains the putative binding sites for Smyd3. Moreover, the mutations at the Smyd3 DNA binding sites, decreased the promoter activity of hTERT and hGata6. Smyd3 siRNA treatment decreased the reporter activity of luciferase reporter plasmids containing hTERT.

## Conclusions

Our results show that Smyd3 represents a crucial epigenetic modulator of hCSC commitment to the myocyte lineage.

